# Influence of Si on the Elevated-Temperature Mechanical and Creep Properties of Al–Cu 224 Cast Alloys during Thermal Exposure

**DOI:** 10.3390/ma17194805

**Published:** 2024-09-29

**Authors:** Kun Liu, Zimeng Wang, Lei Pan, X.-Grant Chen

**Affiliations:** 1 Department of Applied Science, University of Quebec at Chicoutimi, Saguenay, QC G7H 2B1, Canada; kun.liu@uqac.ca (K.L.); zwang8@etu.uqac.ca (Z.W.); 2 Arvida Research and Development Centre, Rio Tinto Aluminum, Saguenay, QC G7S 4K8, Canada; leiray.pan@riotinto.com

**Keywords:** Al–Cu 224 alloy, Si addition, precipitation microstructure, elevated temperature, mechanical properties, creep resistance

## Abstract

The influence of Si content (0.1–0.8 wt.%) on the development of precipitation microstructures and the resultant mechanical and creep properties during thermal exposure, up to 1000 h at 300 °C, in Al–Cu 224 cast alloys, was systematically investigated. The room and elevated temperature yield strength (YS) increased with increasing Si content under the T7 condition, which was attributed to the fact that the Si promoted the precipitation of fine θ′. However, Si increased the coarsening of θ′ during thermal exposure at 300 °C, and the alloys with low Si exhibited a higher YS and creep resistance at elevated temperatures than high Si alloys. The mechanical strength and creep resistance were mainly controlled by the precipitation strengthening of the predominant θ′ phase. Because of the high mechanical strength and creep resistance of the 0.1Si alloy during long-term thermal exposure, the Si level in Al–Cu alloys should be maintained at a low level of 0.1 wt.% for high-temperature applications. The strengthening mechanisms were quantitatively analyzed, based on the characteristics of the precipitate. The predicted YS values under different exposure conditions agreed well with the experimentally measured values.

## 1. Introduction

Because of their high strength-to-mass ratio and adequate-to-excellent mechanical properties, Al–Si cast alloys are widely used in components in the automobile and transportation industries, such as engine blocks and cylinder heads, to reduce the mass of the vehicle and improve engine efficiency [1,2,3]. Improving the efficiency of combustion engines is one of the key methods that can be used in the automobile industry to attain the target 45% reduction in greenhouse gas (GHG) emissions by 2030. This will result in a continual increase in the engine servicing temperature, which is expected to soon range between 250 and 350 °C [4,5,6]. Therefore, stricter requirements have been established for the materials used in the new generation of engines; in particular, their thermal stability at elevated temperatures. Studies have shown that the strength of Al–Si cast alloys exponentially decreases during thermal exposure at elevated temperatures, owing to the high rate of precipitate coarsening [4,5,6,7,8]. The yield strength (YS) of the Al–Si 356 alloy at 315 °C, under the T7 condition, decreased from 165 MPa to 24 MPa after 100 h of exposure [7]. Hence, improving the thermal resistance of Al cast alloys is becoming one of the critical topics in the automobile industry.

Recently, Al–Cu cast alloys have attracted attention owing to their improved thermal resistance at elevated temperatures via various techniques, such as the optimization of chemical compositions [9,10,11,12], the adoption of various heat treatments [13,14,15], as well as microalloying with transition elements [13,15,16,17,18,19,20,21]. For instance, the YS of Al–Cu 224 alloys, comprising optimized Mg content, increased to 186 MPa at 300 °C after T7 treatment; in comparison, the YS of the Mg-free base alloy increased to 109 MPa under the same conditions. Additionally, the YS of the Al–Cu 224 alloy, composed of Mg, stabilized at 125 MPa after 1000 h of exposure at 300 °C [9]. The thermal resistance of the alloy increased by 25% with the combined addition of transition elements, such as Mn, Zr, V, and Ti [16,17]. The mechanical properties and thermal stability of the alloy were further improved by the addition of Sc and the modification of the aging treatment, via the formation of fine and uniformly distributed precipitates [15,19]. Therefore, Al–Cu cast alloys are considered to be one of the most promising materials for fabricating the new generation of engine parts.

In general, the addition of Si to Al–Cu cast alloys, in low quantities, has been performed for a few reasons. First, Si has been shown to improve castability and reduce the tendency for hot tearing [22]. Additionally, Si can improve the mechanical properties of an alloy by neutralizing the negative impact of Fe through the preferential formation of the Chinese script α-Fe over the needle-like β-Fe [23]. However, studies on the influence of Si on the thermal resistance of Al–Cu alloys are limited. A previous study showed that the addition of Mn and Si to an Al–Cu 206 alloy could reduce the thermal stability of precipitation [10]. However, the influence of only the addition of Si remains unclear. Therefore, it is interesting to investigate the effect of varying Si content on the mechanical properties and thermal resistance of Al–Cu cast alloys after long-term thermal exposure at elevated temperatures, which could provide the fundamental scientific knowledge for the development of new engine designs and applications.

In this study, a wide range of Si contents (0.1–0.8 wt.%) were added to the Al–Cu 224 cast alloy to systematically assess the role of Si in the precipitation evolution for varying durations of thermal exposure (up to 1000 h). The mechanical and creep properties of Al–Cu 224 alloys, with a varying amount of Si content, during long-term thermal exposure, were fully evaluated at elevated temperatures. Additionally, the coarsening and strengthening mechanisms were analyzed to elucidate precipitate coarsening and their contributions to the mechanical strength of the alloy during long-term thermal exposure. The experimental results are expected to advance the understanding of the relationship between the microstructure and the mechanical performance of alloys and to provide scientific support and guidance on the design process of Al–Cu 224 alloys for elevated-temperature applications.

## 2. Materials and Methods

Four Al–Cu 224 cast alloys, composed of 0.1, 0.25, 0.5, and 0.8 wt.% Si, were prepared. Commercial Al (99.6 wt.%), Mg (99.9 wt.%), Al-50 wt.%Si, Al-50 wt.%Cu, Al-25 wt.%Mn, and Al-5 wt.%Ti-1 wt.%B master alloys were batched in a crucible, heated in an electric resistance furnace to 780 °C, and then maintained at that temperature for a minimum of 30 min to dissolve all the elements. Thereafter, the alloys were degassed with Ar for 15 min. After degassing, the molten metal was poured into a permanent steel mold, which was preheated to 250 °C. The casting ingots were rectangular, with the dimensions 80 mm × 40 mm × 30 mm. More details about the casting process and sample preparation can be found in previous studies [16,17]. The chemical compositions of the four experimental alloys, analyzed using optical emission spectrometry, are listed in Table 1.

**Figure 1:**
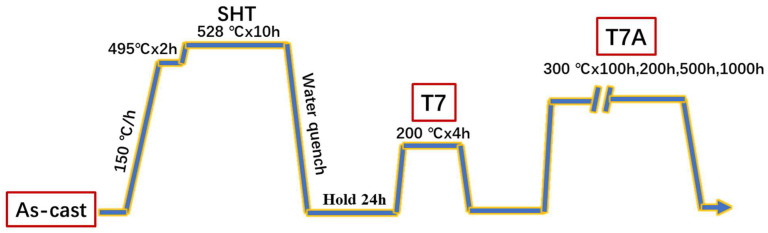
Schematic of the heat treatment processes applied in this study.

The traditional T7 treatment was applied to all the samples after casting, which included a two-step solution treatment (495 °C for 2 h and 528 °C for 10 h and water quenching), followed by an artificial aging treatment (200 °C for 4 h). To investigate the thermal stability of the experimental alloys at elevated temperatures, thermal exposure at 300 °C for varying durations (100, 200, 500, and 1000 h) was applied to the samples, under the T7 condition. These samples, after thermal exposure, were referred to as the T7A samples, identified as “T7Axxx”, where “xxx” stands for the different exposure time in hours. Figure 1 shows the heat treatments applied to the samples.

After heat treatment (T7 and T7A conditions), the compressive YS at room temperature and 300 °C was measured using a Gleeble 3800 universal thermomechanical simulator, at a strain rate of 0.001/s. The test ended at a strain of 0.2. The compressive creep tests were performed on the T7A samples at 300 °C for 90 h, with a constant load of 30 MPa. The cylinder samples for the YS and creep tests were machined to a diameter and height of 10 mm and 15 mm, respectively. A minimum of three tests were performed for each condition, and the average value was determined.

For the microstructural characterization, the evolution of the secondary particles, including the intermetallics and precipitates, were analyzed using optical microscopy (OM), scanning electron microscopy (SEM), and transmission electron microscopy (TEM). The samples observed using OM and SEM were prepared via a standard metallographic procedure. The samples analyzed using TEM were ground and polished to 40–60 μm, then electrochemically twin-jet polished in a solution containing 67 vol.% methanol and 33 vol.% nitric acid, at a temperature from −20 to −30 °C, under 21 V. TEM images were taken in the <001>Al zone-axis condition and analyzed using Clemex V4 image analysis software to assess the various aspects of the precipitates, such as the diameter, number density, and area fraction, based on the statistical analysis of ~1000 precipitates for each condition. Details on the quantification method used are described in previous studies [16,17]. In addition, differential scanning calorimetry (DSC) was utilized to investigate the development of the microstructure after the addition of Si. The heating and cooling rates were set as 10 °C/min.

## 3. Results

### 3.1. Evolution of the Microstructure after the Addition of Varying Si Contents

#### 3.1.1. As-Cast Condition

**Figure 2:**
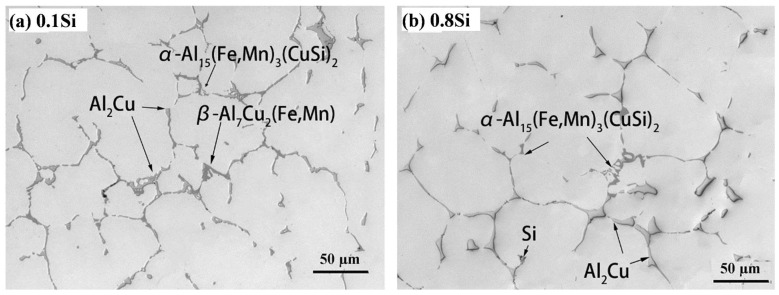
Typical as-cast microstructures: (**a**) 0.1Si, (**b**) 0.8Si alloys.

In general, the as-cast microstructures of the four experimental alloys were similar. These alloys were composed of α-Al, Al_2_Cu, and Fe-rich intermetallic phases (Figure 2), which were identified by an energy-dispersive X-ray spectrometer (EDS) attached to the SEM. However, differences in the Fe-rich intermetallic phases in the four alloys were observed. The variety and morphology of the intermetallics were dependent on the Si content. For example, two types of Fe-rich intermetallics were observed in the 0.1Si alloy (Figure 2a): plate-like Al_7_Cu_2_(Mn, Fe), known as β-Fe, and Chinese script-like Al_15_(Mn, Fe)_3_(Cu, Si)_2_, known as α-Fe. In contrast, only α-Fe was observed in the high Si (0.8Si) alloy (Figure 2b). This indicates that the addition of Si can suppress the transformation of β-Fe, while promoting α-Fe during solidification, which has also been reported in previous studies [10,23]. In addition, the formation of small Si particles was observed in the 0.8Si alloy (Figure 2b).

**Figure 3:**
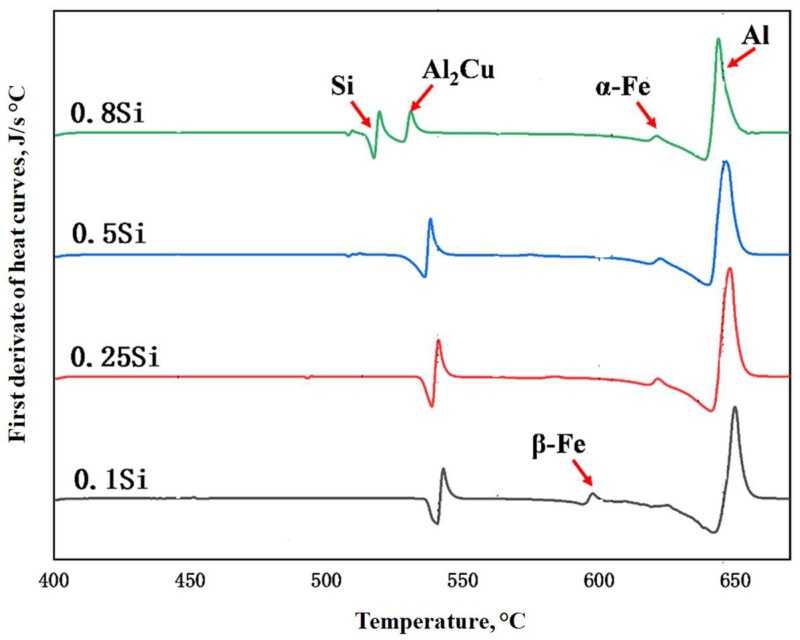
First derivative of DSC heating curves for the as-cast condition.

To confirm the as-cast microstructures, DSC analysis was conducted on the four experimental alloys. The first derivatives of the DSC heating curves are shown in Figure 3. The peaks were indexed according to their formation temperature and the findings in previous studies [23,24]. Moreover, β-Fe and α-Fe were indexed in terms of the 0.1Si alloy, similar to the microstructure shown in Figure 2; additionally, the intensity of the β-Fe peak continually decreased with increasing Si content. Finally, only α-Fe was observed in the alloys with higher Si content (0.5Si and 0.8Si alloys). An Si peak was only detected in the 0.8Si alloy, confirming the presence of Si particles (Figure 2b).

#### 3.1.2. T7 and T7A Conditions

**Figure 4:**
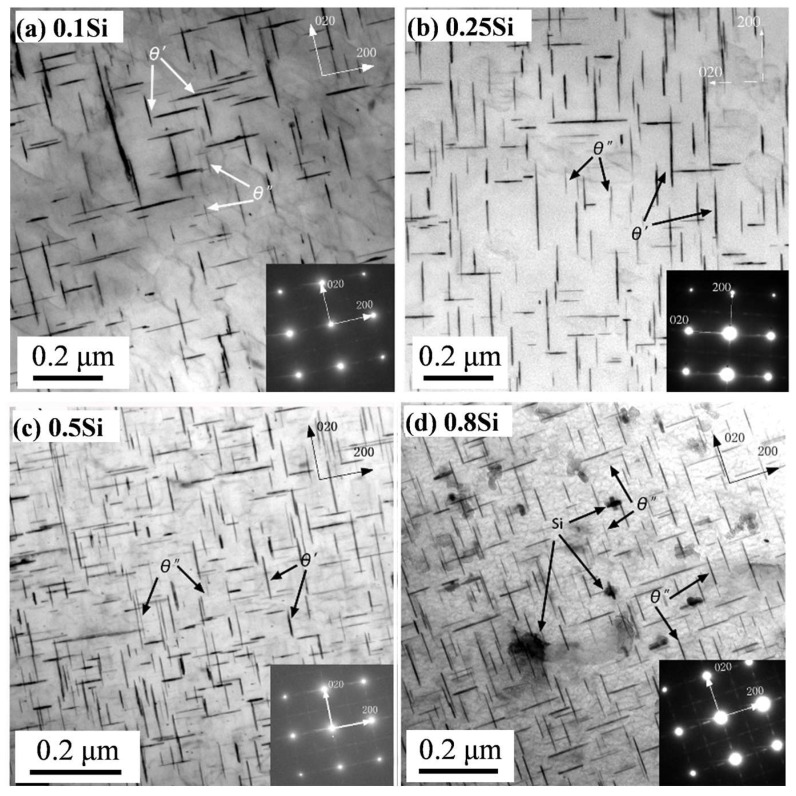
Bright-field TEM images and corresponding SAEDPs of experimental alloys under the T7 condition: (**a**) 0.1Si, (**b**) 0.25Si, (**c**) 0.5Si, and (**d**) 0.8Si alloys.

After T7 heat treatment, the dissolution of the Al_2_Cu intermetallic compound into the Al matrix was almost complete, whereas the β-Fe and/or α-Fe intermetallic phases remained unchanged. The nano-scale precipitates, after aging, were characterized using TEM. Figure 4 shows the bright-field TEM images of the four experimental alloys under the T7 condition. The presence of fine and uniformly distributed θ″ and θ′ precipitates were observed in the experimental alloys; however, the θ′ precipitates were dominant. The addition of Si enhanced the precipitation of the θ′ precipitates, particularly in the higher Si alloys (0.5Si and 0.8Si). The number density (Nv) and volume fraction (f) of θ′ significantly increased with the addition of Si (Table 2). The number density of θ′ in the 0.8Si alloy was twofold greater than that of the 0.1Si alloy (2473 vs. 1236). The diameter (dt) of θ′ marginally increased from 86.4 nm in the 0.1Si alloy to 88.0 nm in the 0.25Si alloy; thereafter, it decreased to 68.0 nm in the 0.8Si alloy. Previous studies [12,25,26,27] have proposed the preferential nucleation of θ′ in Si-rich clusters, because Si can reduce the α-Al/θ′ precipitates’ interfacial energy and stabilize the thermal dynamics of the formation of θ′ precipitates, which could explain the increased number density and smaller size of θ′ with increasing Si levels. The addition of a high amount of Si suppressed the formation of Cu-rich β-Fe, releasing more Cu into the matrix, which could result in a higher volume fraction in terms of the precipitates.

**Figure 5:**
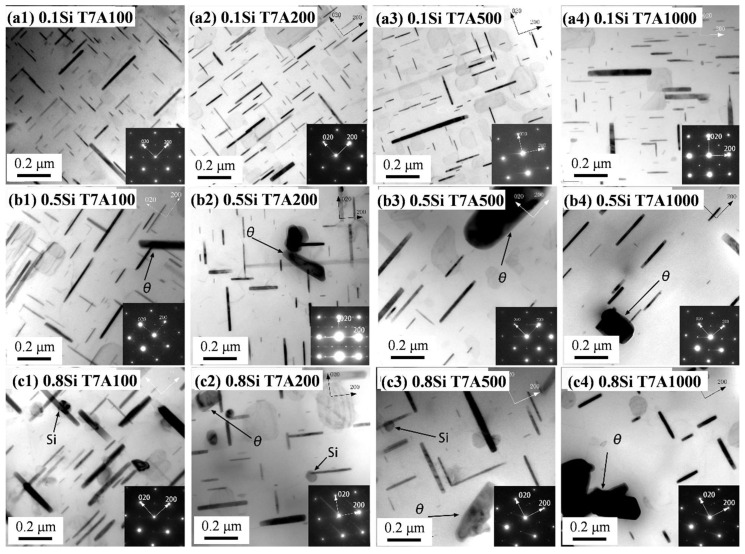
Bright-field TEM images and corresponding SAEDPs showing the coarsening of the θ′ precipitate under varying T7A conditions: (**a1**–**a4**) 0.1Si, (**b1**–**b4**) 0.5Si, and (**c1**–**c4**) 0.8Si alloys.

To investigate the thermal stability of the precipitates, the sample after the T7 treatment was further exposed to a temperature of 300 °C for up to 1000 h (the T7A conditions). Figure 5 shows the bright-field TEM images of the three experimental alloys after being subject to different thermal exposure times, and the quantitative results are summarized in Table 3. Due to the similarity between the 0.1Si and 0.25Si alloys, only the results for the 0.1Si, 0.5Si, and 0.8Si alloys are presented here. It can be found that after the T7A treatments, the θ″ precipitates disappeared, and only the θ′ precipitates were dominant in the precipitation microstructure. With the increasing exposure time, the θ′ precipitates were generally coarsened in all the experimental alloys. Compared with Figure 5 and Table 2, in regard to the T7 condition, the size of the θ′ precipitate is obviously increased, whereas the number density is reduced, especially in alloys with higher Si contents. As shown in Table 2 and Table 3, the diameter of the θ′ precipitate in the 0.1Si alloy increased from 86.4 nm under the T7 condition to 103.1 nm under the T7A1000 condition, whereas it increased from 68 nm under the T7 condition to 116.6 nm, when exposed for 1000 h, in the 0.8Si alloy.

The results show that Si plays a critical role in the coarsening process of θ′ precipitates during thermal exposure. Although the coarsening of the θ′ precipitates in the 0.1Si alloy increased as the duration of exposure increased, no increase in the thickness of the precipitates was observed (Figure 5a1–a4). For example, the thickness of the θ′ precipitates in the 0.1Si alloy remained between 7 and 8 nm, even after 500 h of exposure, whereas the length continuously increased with the increasing duration of the exposure (Table 3). For the alloys with a high amount of Si (Figure 5b1–b4,c1–c4), the length and thickness of the θ′ precipitates increased as the duration of exposure increased. As shown in Table 3, for the 0.8Si alloy, the thickness of the θ′ precipitates in the 0.8Si alloy was 6 nm after being exposed to the T7 condition; however, the thickness increased to 17.8 nm and 22.9 nm after exposures of 500 h and 1000 h, respectively, exhibiting a higher coarsening rate. A coarse equilibrium θ phase was frequently observed in the alloys with a high amount of Si, particularly after extended durations of exposure. In addition, some Si nanoprecipitates were observed to be closely connected to the θ′ precipitates in the 0.8Si alloy (Figure 5c1–c4).

Differences in the evolution of the θ′ precipitates at a given exposure time were observed with varying Si contents. For example, the θ′ precipitates in the 0.1Si alloy after 100 h of exposure were significantly finer than that of the 0.5Si alloy; additionally, the 0.8Si alloy exhibited the highest θ′ value for this duration of exposure. The number density decreased from 498 to 104 μm^−3^ as the Si content increased in terms of the 0.1Si alloy to the 0.8Si alloy (Table 3). Therefore, Si enhanced the formation of θ′ precipitates under the T7 condition; however, θ′ coarsening increased during the exposure to elevated temperature (T7A conditions).

### 3.2. Mechanical Properties of Different Si Alloys

**Figure 6:**
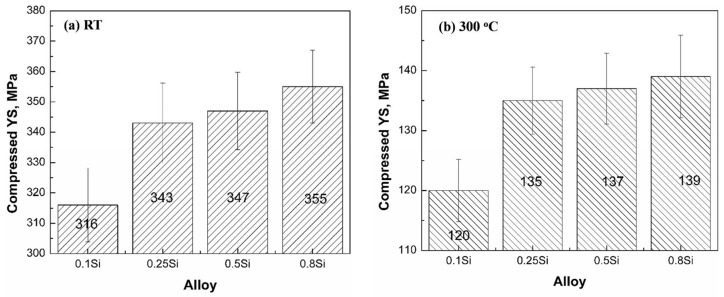
Compressive YS of four experimental alloys under the T7 condition at: (**a**) room temperature and (**b**) 300 °C.

Figure 6 shows the compressive YS of the four experimental alloys at room temperature and 300 °C under the T7 condition. The addition of Si increases the YS at room temperature and 300 °C. At room temperature, the YS increased from 316 MPa in the 0.1Si alloy to 355 MPa in the 0.8Si alloy. When tested at 300 °C, the YS increased from 120 MPa in the 0.1Si alloy to 139 MPa in the 0.8Si alloy. Therefore, 13% and 16% increases in the YS were observed at room temperature and 300 °C, respectively. The increase in the mechanical properties of the alloys with a low amount of Si (0.1Si and 0.25Si alloys) was significantly higher than that of the alloys with a high amount of Si. The YS increased by approximately 9% at room temperature as the Si content in the alloy increased from 0.1 to 0.25Si, whereas the YS increased by approximately 2% as the Si content increased from the 0.5 to the 0.8Si alloy. All the changes in the YS were associated with the evolution of the precipitates. The number density and fraction of the precipitates increased, whereas the size decreased, with the increasing Si content (Figure 4 and Table 2). This explained the tendency for improved mechanical properties under the T7 condition for the different Si content alloys. However, the YS of each alloy significantly decreased at 300 °C because of the softening of the Al matrix at elevated temperatures. In general, the YS value at 300 °C was approximately 40% of the YS value at room temperature.

**Figure 7:**
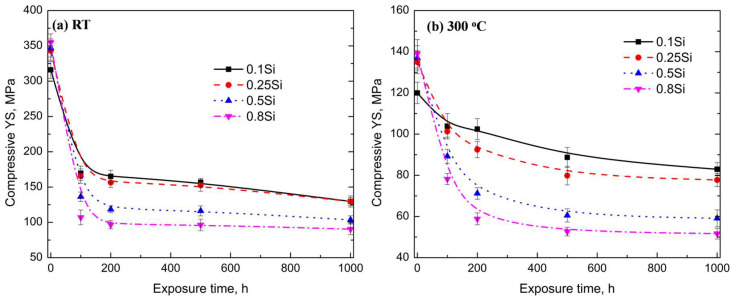
Compressive YS of experimental alloys as a function of the duration of exposure (T7A conditions) at: (**a**) room temperature and (**b**) 300 °C.

The long-term thermal stability of the mechanical properties of high-temperature resistant Al alloys is a critical aspect. Figure 7 shows the compressive YS of the experimental alloys at room temperature and 300 °C after varying exposure durations (up to 1000 h). Because of the coarsening process of θ′ precipitates during thermal exposure (Figure 5), the compressive YS decreased with the increasing duration of exposure at room temperature and 300 °C for all the alloys. All the YS values exponentially decreased during the early stages of thermal exposure (100–200 h). Thereafter, the rate of reduction decreased or plateaued with prolonged exposure. For example, the YS of the 0.5Si alloy at 300 °C decreased from 137 MPa under the T7 condition to 71 MPa under the T7A200 condition (Figure 7b); thereafter, the YS value decreased to 59 MPa under the T7A1000 condition.

The pattern of the YS reduction varied with the variation in Si content. In general, the rate of reduction in terms of the mechanical properties was lower in the alloys with a low amount of Si than in the alloys with a high amount of Si. For example, the YS of the 0.1Si alloy decreased from 316 MPa under the T7 condition to 170 MPa under the T7A100 condition (a 46% reduction after 100 h of thermal exposure) at room temperature. The value of this change was significantly higher in the 0.8Si alloy, reducing by 70% at room temperature (from 355 MPa to 107 MPa). A similar trend in the YS reduction was observed at 300 °C. After 200 h of exposure, 15 and 55% reductions in YS were observed for the 0.1Si (from 120 to 102 MPa) and 0.8Si alloys (from 139 to 58 MPa), respectively. The higher rate of YS reduction in the alloys with a higher amount of Si after thermal exposure resulted in an opposing relationship between the mechanical properties and the Si content. The YS increased with the increasing Si content under the T7 condition; however, the YS decreased with the increasing Si content under the T7A conditions.

### 3.3. Creep Resistance of Various Si Alloys

**Figure 8:**
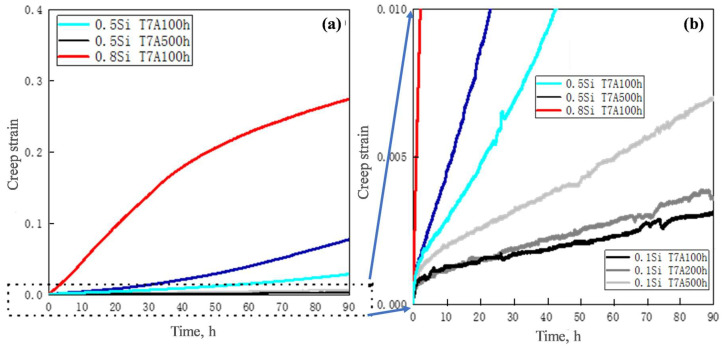
Typical creep curves of experimental alloys under varying thermal exposure conditions at creep strains ranging from: (**a**) 0 to 0.4 and (**b**) 0 to 0.01.

The properties of the experimental alloys at elevated temperatures after thermal exposure were evaluated via compression creep tests at 300 °C, under a constant load of 30 MPa. The typical creep curves are shown in Figure 8. The minimum creep rate and total creep strain of the experimental alloys under different thermal exposure conditions are listed in Table 4. The minimum creep rates are calculated from the creep curves during the quasi-steady stage [17], which is used in combination with the total creep strain to characterize the creep resistance of alloys.

During creep deformation at an elevated temperature, the creep strain of the 0.1Si alloy under three conditions increased rapidly in the first few hours. Thereafter, this value gradually increased with the creep time, which showed that the creep was entering the quasi-steady stage. The minimum creep rate and total strain increased with the increasing exposure time (Figure 8a and Table 4). The T7A500h sample exhibited the highest minimum creep rate (1.6 × 10^−8^ s^−1^) and total strain (0.007).

The rate of increase in the creep strain of the 0.5Si alloy under all three conditions was significantly higher than that of the 0.1Si alloy, even during the first 10 to 30 h (Figure 8b), resulting in higher minimum creep rate and total strain (Table 4). The minimum creep rate and total creep strain of the 0.5Si T7A100h sample were the lowest; however, the 0.5Si T7A500h sample exhibited the highest minimum creep rate and total creep strain. Compared with the 0.1Si alloy, the lowest minimum creep rate and total strain for the 0.5Si T7A100h sample were still higher than those of the 0.1Si T7A500h sample, in the same creep conditions. In addition, the minimum creep rate and total creep strain of the 0.5Si alloy increased at a significantly higher rate than those of the 0.1Si alloy for the prolonged thermal exposure. This occurred because of the larger size of the θ′ precipitates and the higher rate of coarsening in the 0.5Si alloy during thermal exposure than the 0.1Si alloy (Figure 5 and Table 3), resulting in insufficient strengthening of the θ′ precipitates in the Al matrix, which acted as dislocation movement barriers during creep deformation.

The 0.8Si alloy exhibited poor creep resistance. The creep strain of the 0.8Si T7A100h sample increased at a significantly higher rate, even than that of the 0.5Si T7A500h sample (Figure 8a). The minimum creep rate and total strain of the 0.8Si alloy after 100 h of exposure were as high as 4.68 × 10^−7^ s^−1^ and 0.273, respectively, which were significantly higher than those of the 0.1Si and 0.5Si alloys after 500 h of exposure. It is evident that the elevated-temperature creep resistance in Al–Cu 224 alloys significantly deteriorated with the increasing Si content.

In brief, the 0.1Si alloy exhibited higher mechanical strength and improved creep resistance during long-term thermal exposure than those of the other experimental alloys. Therefore, the Si content in Al–Cu alloys should be maintained at a low level of 0.1 wt.% for high-temperature applications.

## 4. Discussion

### 4.1. Evolution of the Microstructure of Various Si Alloys 

Si plays distinct roles in the evolution of precipitates and the improvement of mechanical properties in alloys under T7 and T7A conditions. Si promoted the formation of θ′ precipitates under the T7 condition; additionally, the number density and volume fraction of the θ′ precipitates increased with the increasing Si content (Figure 4 and Table 2). However, Si accelerated the coarsening of θ′ precipitates during thermal exposure with increasing exposure durations. Increasing the Si content in the alloy increased the diameter and thickness of the θ′ precipitates; however, the number density decreased under the same thermal exposure (Figure 5 and Table 3).

The enhancement of θ′ precipitation by increasing the Si content was attributed to the interaction between Si and θ′ during the T7 treatment. Si clusters can act as nucleation sites for θ′ precipitates, owing to the lower strain energy between θ′ precipitates and pre-existing Si clusters [12,25,26,27]. Additionally, Si can reduce the α-Al/θ′ interfacial energy, thereby dynamically stabilizing θ′ precipitates [12,25,26,27]. The Si nanoprecipitates in the 0.8Si alloy under the T7 condition were closely connected with the θ′ precipitates (Figure 4), indicating that the nucleation effect of the Si clusters/nanoprecipitates on the formation of θ′ precipitates likely occurred. The addition of high Si content could also introduce more quenched-in vacancies after solution treatment, which can act as the nuclei for θ′ precipitates during subsequent aging [11,28,29]. Therefore, based on all the possible interactions between Si and θ′ precipitates, the addition of Si likely enhances θ′ precipitation under the T7 condition.

During long-term thermal exposure, the coarsening rate of the θ′ precipitates in alloys with a high amount of Si was higher than that of alloys with a low amount of Si, resulting in coarser θ′ precipitates with lower number densities as the Si content increases. Consequently, the mechanical and creep properties decreased under the T7A conditions (Figure 7 and Figure 8). Therefore, the coarsening rate of θ′ precipitates is the critical parameter for applications at elevated temperatures. The thermodynamically driven coarsening of θ′ precipitates can be expressed by the following equation [30]:

(1) $${\bar{r}}^{n}-{\bar{r}}_{0}^{n}=k\left(t-t_{0}\right)$$

where *r* is the mean radius of the θ′ precipitates at time *t*; *r*_0_ is the mean radius of the θ′ precipitates at starting time *t*_0_, which corresponded to the T7 condition in this study; *t* is the thermal exposure time; *k* is the constant for the coarsening rate of the θ′ precipitates; and *n* is the temporal exponent, which is accepted as a value of 3, based on previous studies [31,32].

**Figure 9:**
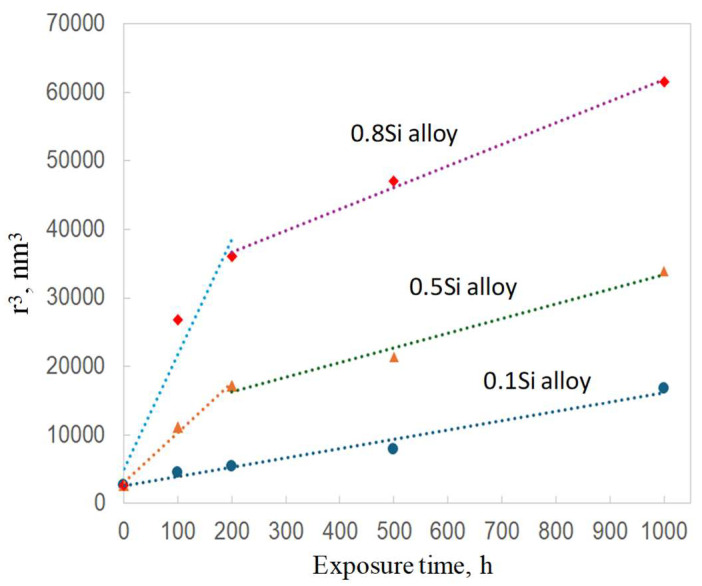
Statistical results on the coarsening evolution of θ′ precipitates during thermal exposure (0 h stands for the T7 condition).

The coarsening of θ′ precipitates as a function of the thermal exposure time, in three alloys, is shown in Figure 9. The *k* value of the experimental alloys was calculated using Equation (1), based on the quantitative results in Table 2 and Table 3; the results are summarized in Table 5. The coarsening rate of the θ′ precipitates in the 0.1Si alloy was constant during the thermal exposure (Figure 9), whereas that of the 0.5Si and 0.8Si alloys can be divided in two stages, namely the coarsening rate during the first stage (0 to 200 h) was higher than that during the second stage (200 to 500 h). In general, the *k* value of the 0.1Si alloy was significantly lower than that of the 0.5Si and 0.8Si alloys at both stages (Table 5). This showed that the rate of reduction in terms of the mechanical and creep properties of the alloys with a high amount of Si were higher than those of the alloys with a low amount of Si (Figure 7). The *k* value of the 0.1Si alloy (13.8) remained constant, whereas that of the 0.8Si alloy (167) was high during the first stage of thermal exposure (Table 5) and decreased to 31 during the second stage of thermal exposure. Therefore, the *k* values of the 0.8Si alloy were 12-fold and 2.25-fold higher than that of the 0.1Si alloy.

The lower reduction in the mechanical properties of the alloys with a high amount of Si after exposures of 200–500 h was likely due to the reduction in the *k* values during the second stage (Figure 7). In addition, the *k* values during each stage for the 0.5Si alloy were lower than those of the 0.8Si alloys (73 vs. 167 and 21 vs. 31, Table 5). This resulted in the 0.5Si alloy exhibiting higher mechanical and creep properties than the 0.8Si alloy during thermal exposure.

**Figure 10:**
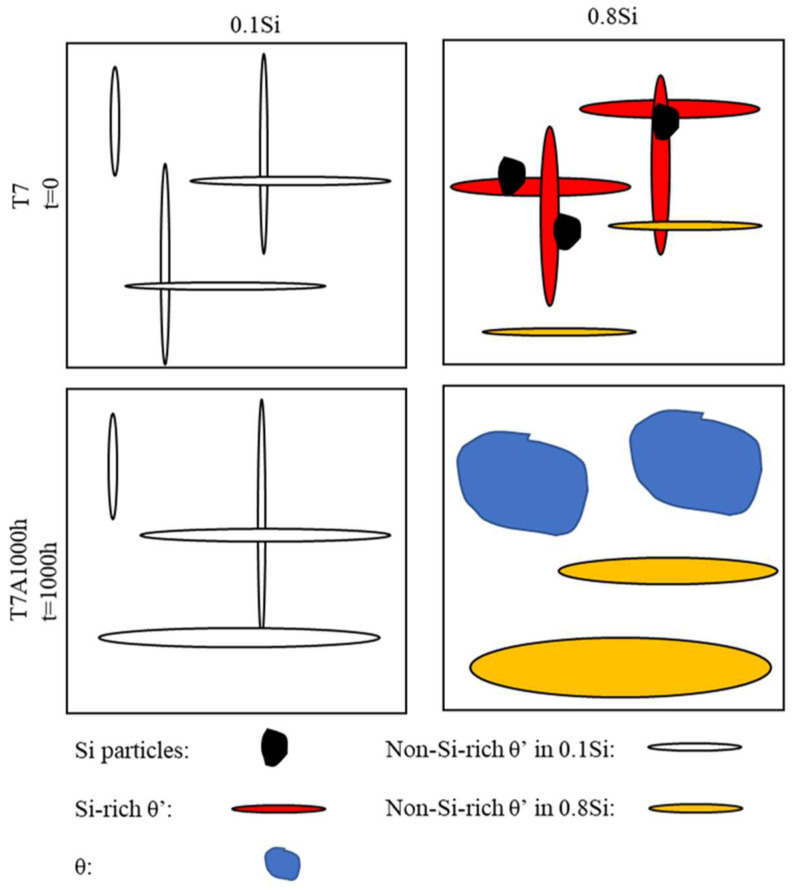
Schematic of the coarsening of θ′ precipitates in Al–Cu 224 alloys.

**Figure 11:**
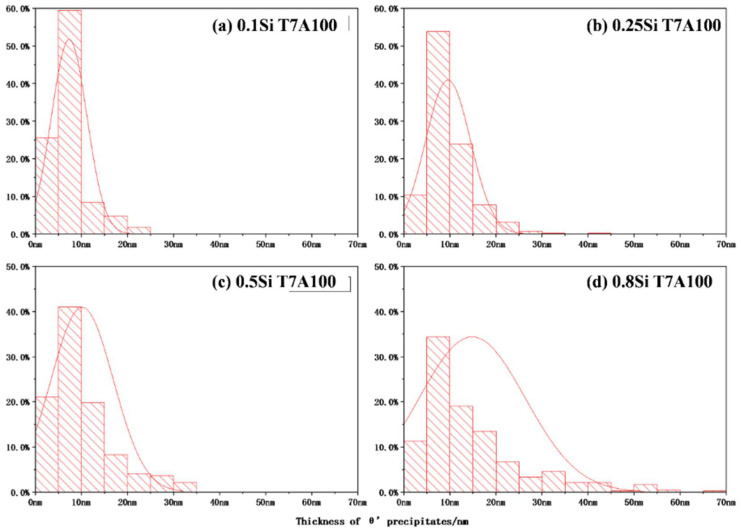
Distribution of the thickness of θ′ precipitates in different alloys under the T7A100h condition.

The two-stage coarsening rates of the θ′ precipitates in the alloys with a high amount of Si during thermal exposure can be explained by the interaction between the Si and the θ′ precipitates. In this study, two coarsening mechanisms are proposed (Figure 10). First, the coarsening of the precipitates via a conventional mechanism resulted in the consumption of the dissolvable, smaller precipitates [33,34], which could be applied to all experimental alloys. However, the number density of the θ′ precipitates increased with the increasing Si content (Figure 3 and Table 2), resulting in lower diffusion distances between the precipitates and a higher coarsening rate. The higher the Si content, the higher the number density of the θ′ precipitates and the higher the coarsening rate were. The second mechanism is related to the Si clusters/nanoparticles that are connected to the θ′ precipitates in the alloys with a high amount of Si. The high Si alloys presumably contain two types of θ′ precipitates, Si-rich θ′ and Si-deficient θ′ precipitates, caused by the nonuniform distribution of Si clusters/nanoparticles. Owing to the high diffusion rate of Si, the coarsening of Si-rich θ′ precipitates, which were in the proximity of the Si clusters/nanoparticles, would generally occur at a higher rate than that of Si-deficient θ′ precipitates. Additionally, some Si-rich θ′ precipitates transfer to the coarse equilibrium θ phase. The segregation of Si solutes to form θ′ precipitates was greater in the alloys with a high amount of Si than that of the alloys with a low amount of Si; therefore, the coarsening of the precipitates occurred at a higher rate in the former than that in the latter. Thus, the size distribution of θ′ precipitates in alloys with a high amount of Si will likely be wider than that of alloys with a low amount of Si. This was confirmed by the thickness distribution of the different alloys during thermal exposure (Figure 11), which was identified from the image analysis results of the precipitates under various conditions in the TEM images. The θ′ precipitate thickness of the 0.1Si alloy after 100 h of exposure (T7A100) was distributed within a narrow range (Figure 11a); additionally, a thickness of approximately 80% of the θ′ precipitates was similar to the average value. The θ′ thickness increased and was distributed over a wider range as the Si content increased, indicating that thick θ′ precipitates were present in the alloys with a high amount of Si owing to the Si segregation and the resulting high coarsening rates. The thickness of most of the 0.8Si alloy’s θ′ precipitates ranged from 5 to 40 nm (Figure 11d), whereas that of the alloys with a low amount of Si ranged from 5 to 15 nm (0.1Si and 0.2Si alloys, Figure 11a,b). Increasing the duration of thermal exposure reduced the available source of coarsening in the alloys with a high amount of Si, such as the dissolvable, small size of the θ′ precipitates (Figure 5), leading to the second stage exhibiting lower coarsening rates than that of the first stage. In contrast, the variation in the thickness of θ′ precipitates in the 0.1Si alloy was lower than that of the alloys with a high amount of Si (Table 3), resulting in a constant coarsening rate in the alloys with a low amount of Si.

### 4.2. Strengthening Mechanisms during Thermal Exposure

The main strengthening mechanism in terms of heat-treatable age-hardening Al–Cu alloys is precipitation strengthening. Therefore, the mechanical strength at room temperature is generally controlled by the size, type, number density, and volume fraction of nanoscale θ″/θ′ precipitates. The following strength models were implemented to explain and predict the strengthening contributions under different thermal exposure, based on the microstructural parameters of the precipitates.

Based on the coherence with the α-Al matrix and its shearability, the θ″ precipitate has been shown to contribute to the critical resolved shear stress (CRSS) mainly through coherency strengthening and interfacial strengthening. Their contribution to mechanical strength is expressed below [20]:

(2) $$∆\tau_{\theta^{\prime \prime }}=4.1\cdot G\left|\varepsilon^{\frac{3}{2}}\right|\cdot {\left[\frac{fd_{t}}{2b}\right]}^{\frac{1}{2}}$$

For semi-coherent and non-shearable θ′ precipitates, their contribution to CRSS can be described by the Orowan bypassing mechanism; the corresponding effective equation is given by [3,20]:

(3) $$∆\tau_{\theta^{\prime }}=\frac{Gb}{2\pi \sqrt{1-v}}\cdot \frac{1}{\frac{1.23\;\times \;1.03}{\sqrt{N_{v}d_{t}}}-\frac{\pi d_{t}}{8}-1.061t_{t}}\cdot \ln\frac{\sqrt{d_{t}t_{t}}}{b}\;$$

where *G* is the shear modulus of the α-Al matrix (25.4 GPa) [20]; *ε* is the lattice strain (0.006) [20]; *b* is the Burgers vector (0.284 nm) [20]; *v* is the Poisson ratio (0.33 for face-centered cubic metals) [20]; and *f*, *dt*, and *Nv* are the volume fraction, diameter, and number density of the precipitates, respectively.

To develop an overall level of CRSS contribution by these two types of precipitates, Equation (4) is given by [20] as follows:

(4) $${∆\tau }^{q}={\left(∆\tau_{\theta^{\prime }}\right)}^{q}+{\left(∆\tau_{\theta \prime \prime }\right)}^{q}$$

where ∆*τ* is the combined CRSS contribution of θ″ and θ′ precipitates and *q* is an exponent that is accepted as two in this study [17].

Using the CRSS contribution, the increase in YS due to the formation of precipitates can be determined by Equation (5) [20]:

(5) $$∆\sigma_{p}=M\cdot ∆\tau$$

where *M* is the Taylor factor (3.06) [20].

Based on the quantitative parameters of the precipitates (Table 2 and Table 3), the contribution of the precipitates to the mechanical strength at room temperature under varying thermal exposure conditions was calculated; the results are summarized in Table 6.

Generally, the overall YS of polycrystalline alloys is influenced by other strengthening components, such as the grain boundary and solid solution. Therefore, the YS of experimental Al–Cu alloys can be estimated using Equation (6) [35,36]:

(6) $$\sigma_{y}=\sigma_{0}+\sigma_{gb}+\sigma_{ss}{+\sigma }_{p}$$

where *σ_0_* is the original YS of the pure Al matrix, *σ_gb_* is the contribution from the grain boundary strengthening, *σ_ss_* is the contribution of the solid solution strengthening, and *σ_p_* is the contribution of the precipitates.

**Figure 12:**
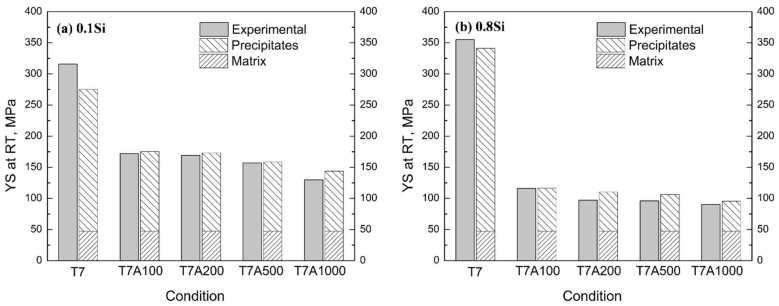
The theoretical and experimental YS values at room temperature during thermal exposure: (**a**) 0.1Si alloy and (**b**) 0.8Si alloy.

In this study, the YS value of the pure Al matrix was accepted as 34 MPa for the 1100-O alloy [17]. The grain boundary strengthening was estimated using the Hall–Petch equation (~10 MPa), at a grain size of 40–50 µm, for the experimental alloys. Regarding solid solution strengthening, supersaturated Cu solutes, mostly precipitated from Al, and the solubility of Cu at room temperature, after the T7 and T7A treatments, was low (~0.1 wt.% according to Al–Cu binary phase diagram), because of the complete precipitation of θ″ and θ′. Hence, the calculated *σ_ss_* was ~3 MPa [37,38]. For simplicity, the contributions from the Al matrix, in combination with the grain size and solid solution effects, were assumed to be the same for all the experimental alloys. The summation of all these contributions provided a value of 47 MPa. The total YS was calculated based on the data shown in Table 6. Figure 12 shows the comparison between the calculated and experimental YS values, using the 0.1Si and 0.8Si alloys as examples.

In general, the calculated YS agreed with the experimentally measured YS for all the experimental alloys under the T7 and T7A conditions (Figure 12). For example, the calculated and experimental YS values for the 0.1Si alloy at T7A200 were 165 MPa and 173 MPa, respectively, whereas those of the 0.8Si alloy corresponded to 109 MPa and 99 MPa. Based on the precipitates in Table 5 and the theoretical and experimental YS values, the θ′ precipitate was the predominant strengthening phase, along with the Orowan bypassing mechanism, during thermal exposure in all the experimental alloys. For example, because a minimal quantity of θ″ precipitates was observed, θ′ precipitation strengthening accounted for 80% of the total YS of the 0.1Si alloy under the T7 condition. During thermal exposure, although its contribution weakens due to coarsening, the precipitates still contribute ~67% to the total YS after 1000 h of thermal exposure. A similar tendency was observed in the 0.8Si alloy. The contribution of θ′ precipitates to the total YS of the 0.8Si alloy was ~85% under the T7 condition (Figure 12b); however, this decreased to ~50% after 1000 h of thermal exposure, owing to its significantly higher coarsening rate.

As mentioned above, Si enhanced the formation of θ′ precipitates under the T7 condition, resulting in the augmentation of the number density and volume fraction of θ′ precipitates in the alloys with a high amount of Si. Therefore, the YS of the alloys with a high amount of Si is expected to be higher than that of the Si alloys with a low amount of Si under the T7 condition (confirmed by Figure 12). In contrast, Si accelerated the coarsening of θ′ precipitates during thermal exposure at an elevated temperature, leading to a lower number density of θ′ precipitates in the alloys with a high amount of Si. As the main θ′ precipitate strengthening contribution decreased with the increasing Si content, the YS of the 0.8Si alloy was assumed to be lower than that of the 0.1Si alloy (prediction in Table 5 and Figure 12).

In general, the YS of the Al alloy at 300 °C was significantly lower than that at room temperature because of the softening of the Al matrix at elevated temperatures, in which the shear modulus (G) of the α-Al matrix reduced from 28.0 GPa at 20 °C to 21.2 GPa at 300 °C [17]. Appling Equations (3)–(5) at 300 °C, the trend in the YS changes induced by the varying Si content was the same as the trend at room temperature, because the elevated-temperature YS was mainly controlled by the microstructural parameters of the θ′ precipitates.

## 5. Conclusions

In this study, the distinct influence of Si additions on the evolution of the microstructure and resultant mechanical and creep properties during long-term thermal exposure, at 300 °C, in Al–Cu 224 cast alloys was, for the first time, systematically investigated. The results are summarized as follows:

(1). Under the T7 condition, the addition of Si enhanced the formation of θ′ precipitates, leading to enhanced mechanical strength with increasing Si content;
(2). During thermal exposure (T7A conditions), Si accelerated the coarsening of θ′ precipitates with increasing exposure durations. The alloys with a high amount of Si exhibited significantly higher coarsening rates of θ′ precipitates compared to the alloys with a low amount of Si, resulting in a significant reduction in their mechanical strength and creep resistance;
(3). The 0.1Si alloy exhibited the highest mechanical strength and best creep resistance during long-term thermal exposure among the four experimental alloys. For high-temperature applications, the Si content in Al–Cu alloys should remain as low as 0.1 wt.%;
(4). The θ′ precipitates was the predominant strengthening phase in Al–Cu 224 alloys; the mechanical strength was mainly controlled by θ′ precipitation strengthening. The proper application of the Orowan bypass mechanism and its strengthening equation predicted the YS values during thermal exposure, which agreed with the experimentally measured values.

## Figures and Tables

**Table 1:** Chemical composition of experimental alloys (wt.%).

Alloys	Cu	Si	Fe	Mg	Mn	Ti	Al
0.1Si	4.24	0.13	0.17	0.11	0.32	0.098	Bal
0.25Si	4.13	0.26	0.17	0.11	0.31	0.106	Bal
0.5Si	4.24	0.48	0.17	0.11	0.33	0.098	Bal
0.8Si	4.36	0.77	0.19	0.10	0.30	0.098	Bal

**Table 2:** Quantification results for θ′ and θ″ precipitates for the T7 condition.

Alloy/Precipitates		Diameter (dt)/nm	Thickness/nm	Nv/μm^−3^	f/%
0.1Si	θ″	30.2 ± 10.2	3.2 ± 1.5	571	0.11
	θ′	86.4 ± 22.8	4.9 ± 2.3	1236	3.47
0.25Si	θ″	26.4 ± 11.5	3.0 ± 1.2	524	0.09
	θ′	88.0 ± 25.2	5.6 ± 3.1	1416	4.66
0.5Si	θ″	34.2 ± 11.8	3.3 ± 1.7	210	0.06
	θ′	76.4 ± 31.0	6.3 ± 2.8	1948	5.88
0.8Si	θ″	34.0 ± 10.4	3.0 ± 1.8	424	0.11
	θ′	68.0 ± 31.8	6.2 ± 2.1	2473	5.87

**Table 3:** Quantification results for θ′ precipitates after different thermal exposure times (T7A conditions).

Condition/Alloy		Diameter (dt)/nm	Thickness/nm	Nv/μm^−3^	f/%
T7A100h	0.1Si	85.8 ± 21.5	7.3 ± 3.1	498	2.14
	0.5Si	104.7 ± 18.8	10.3 ± 2.8	239	2.67
	0.8Si	111.5 ± 24.2	14.8 ± 3.5	104	2.93
T7A200h	0.1Si	88.1 ± 15.9	7.8 ± 2.9	458	1.76
	0.5Si	109.5 ± 20.1	12.5 ± 3.2	184	2.03
	0.8Si	123.7 ± 18.8	16.2 ± 3.8	75	2.38
T7A500h	0.1Si	98.6 ± 20.3	8.4 ± 2.4	396	1.75
	0.5Si	116.7 ± 19.8	12.9 ± 2.8	98	1.45
	0.8Si	148.1 ± 21.1	17.8 ± 3.1	51	1.54
T7A1000h	0.1Si	103.1 ± 18.8	12.8 ± 4.1	209	1.48
	0.5Si	134.8 ± 21.3	15.8 ± 3.4	58	1.37
	0.8Si	116.6 ± 21.8	22.9 ± 3.6	48	1.19

**Table 4:** Minimum creep rate and total creep strain of experimental alloys under different thermal exposure conditions.

Alloy	Condition	Minimum Creep Rate, s^−1^	Total Creep Strain, 90 h
0.1Si	T7A100h	5.86 × 10^−9^	0.0031
	T7A200h	9.53 × 10^−9^	0.0041
	T7A500h	1.6 × 10^−8^	0.0073
0.5Si	T7A100h	2.81 × 10^−8^	0.0124
	T7A200h	4.69 × 10^−8^	0.030
	T7A500h	1.05 × 10^−7^	0.047
0.8Si	T7A100h	4.68 × 10^−8^	0.273

**Table 5:** Coarsening rate constant k of different experimental alloys.

Alloys	Exposure Time	*k* in nm^3^/h	R^2^
0.1Si	0~1000 h	13.8	95.0
0.5Si	0~200 h	73.0	98.9
	200~1000 h	21.5	93.8
0.8Si	0~200 h	167.0	93.5
	200~1000 h	31.3	99.5

**Table 6:** Contribution of precipitates to YS increments in alloys at RT, in MPa.

Alloy	T7	T7A100	T7A200	T7A500	T7A1000
	θ″ + θ′	θ′	θ′	θ′	θ′
0.1Si	228.1	128.3	126.2	111.4	96.8
0.5Si	285.1	103.1	81.5	68.1	57.9
0.8Si	294.2	69.3	63.1	59.1	48.7

## Data Availability

The data that support the findings of this study are available from the corresponding author upon reasonable request.

## References

[1] Molina R., Amalberto-Teksid P., Rosso M. (2011). Mechanical characterization of aluminium alloys for high temperature applications Part 1: Al-Si-Cu alloys. Metall. Sci. Technol..

[2] Javidani M., Larouche D. (2014). Application of cast Al–Si alloys in internal combustion engine components. Int. Mater. Rev..

[3] Roy S., Allard L.F., Rodriguez A., Watkins T.R., Shyam A. (2017). Comparative Evaluation of Cast Aluminum Alloys for Automotive Cylinder Heads: Part I—Microstructure Evolution. Metall. Mater. Trans. A.

[4] Farkoosh A.R., Chen X.G., Pekguleryuz M. (2015). Interaction between molybdenum and manganese to form effective dispersoids in an Al–Si–Cu–Mg alloy and their influence on creep resistance. Mater. Sci. Eng. A.

[5] Farkoosh A.R., Grant Chen X., Pekguleryuz M. (2015). Dispersoid strengthening of a high temperature Al–Si–Cu–Mg alloy via Mo addition. Mater. Sci. Eng. A.

[6] Jin L., Liu K., Chen X.G. (2020). Evolution of dispersoids and their effects on elevated-temperature strength and creep resistance in Al-Si-Cu 319 cast alloys with Mn and Mo additions. Mater. Sci. Eng. A.

[7] Kaufman J.G. (1999). Properties of Aluminum Alloys Tensile, Creep, and Fatigue Data at High and Low Temperatures.

[8] Chen S., Liu K., Chen X.G. (2019). Precipitation behavior of dispersoids and elevated-temperature properties in Al–Si–Mg foundry alloy with Mo addition. J. Mater. Res..

[9] Rakhmonov J., Liu K., Pan L., Breton F., Chen X.G. (2020). Enhanced mechanical properties of high-temperature-resistant Al–Cu cast alloy by microalloying with Mg. J. Alloys Compd..

[10] Rakhmonov J., Liu K., Chen X.G. (2020). Effects of Compositional Variation on the Thermal Stability of θ′-Al_2_Cu Precipitates and Elevated-Temperature Strengths in Al-Cu 206 Alloys. J. Mater. Eng. Perform..

[11] Shyam A., Roy S., Shin D., Poplawsky J.D., Allard L.F., Yamamoto Y., Morris J.R., Mazumder B., Idrobo J.C., Rodriguez A. (2019). Elevated temperature microstructural stability in cast AlCuMnZr alloys through solute segregation. Mater. Sci. Eng. A.

[12] Shin D., Shyam A., Lee S., Yamamoto Y., Haynes J.A. (2017). Solute segregation at the Al/θ′-Al_2_Cu interface in Al-Cu alloys. Acta Mater..

[13] Gao Y.H., Cao L.F., Kuang J., Zhang J.Y., Liu G., Sun J. (2020). Assembling dual precipitates to improve high-temperature resistance of multi-microalloyed Al–Cu alloys. J. Alloys Compd..

[14] Mondol S., Kumar S., Chattopadhyay K. (2019). Effect of thermo-mechanical treatment on microstructure and tensile properties of 2219ScMg alloy. Mater. Sci. Eng., A.

[15] Gao Y.H., Cao L.F., Yang C., Zhang J.Y., Liu G., Sun J. (2019). Co-stabilization of θ′-Al_2_Cu and Al_3_Sc precipitates in Sc-microalloyed Al–Cu alloy with enhanced creep resistance. Mater. Today Nano.

[16] Hu P., Liu K., Pan L., Chen X.G. (2023). Effects of individual and combined additions of transition elements (Zr, Ti and V) on the microstructure stability and elevated-temperature properties of Al–Cu 224 cast alloys. Mater. Sci. Eng. A.

[17] Li D., Liu K., Rakhmonov J., Chen X.G. (2021). Enhanced thermal stability of precipitates and elevated-temperature properties via microalloying with transition metals (Zr, V and Sc) in Al–Cu 224 cast alloys. Mater. Sci. Eng. A.

[18] Yang C., Cao L., Gao Y., Cheng P., Zhang P., Kuang J., Zhang J., Liu G., Sun J. (2020). Nanostructural Sc-based hierarchy to improve the creep resistance of Al–Cu alloys. Mater. Des..

[19] Rouxel B., Ramajayam M., Langan T.J., Lamb J., Sanders P.G., Dorin T. (2020). Effect of dislocations, Al_3_(Sc,Zr) distribution and ageing temperature on θ′ precipitation in Al-Cu-(Sc)-(Zr) alloys. Materialia.

[20] Mondol S., Makineni S.K., Kumar S., Chattopadhyay K. (2018). Enhancement of High Temperature Strength of 2219 Alloys Through Small Additions of Nb and Zr and a Novel Heat Treatment. Metall. Mater. Trans. A.

[21] Kumar Makineni S., Sugathan S., Meher S., Banerjee R., Bhattacharya S., Kumar S., Chattopadhyay K. (2017). Enhancing elevated temperature strength of copper containing aluminium alloys by forming L12 Al3Zr precipitates and nucleating θ″ precipitates on them. Sci. Rep..

[22] Li W., Cui S., Han J., Xu C. (2006). Effect of Silicon on the casting properties of Al-5.0% Cu alloy. Rare Met..

[23] Liu K., Cao X., Chen X.G. (2011). Solidification of iron-rich intermetallic phases in Al-4.5Cu-0.3Fe cast alloy. Metall. Mater. Trans. A.

[24] Lin B., Xu R., Li H., Zhang W. (2018). Formation of Fe-rich intermetallics in Al–5.0Cu–0.5 Fe alloys with different Mn additions. Mater. Sci. Technol..

[25] Mitlin D., Morris J.W., Radmilovic V. (2000). Catalyzed precipitation in Al-Cu-Si. Metall. Mater. Trans. A.

[26] Biswas A., Siegel D.J., Wolverton C., Seidman D.N. (2011). Precipitates in Al–Cu alloys revisited: Atom-probe tomographic experiments and first-principles calculations of compositional evolution and interfacial segregation. Acta Mater..

[27] Shower P., Poplawsky J., Bahl S., Shyam A. (2021). The role of Si in determining the stability of the θ′ precipitate in Al-Cu-Mn-Zr alloys. J. Alloys Compd..

[28] Wolverton C. (2007). Solute–vacancy binding in aluminum. Acta Mater..

[29] Stroev A.Y., Gorbatov O.I., Gornostyrev Y.N., Korzhavyi P.A. (2018). Solid solution decomposition and Guinier-Preston zone formation in Al-Cu alloys: A kinetic theory with anisotropic interactions. Phys. Rev. Mater..

[30] Baldan A. (2002). Review Progress in Ostwald ripening theories and their applications to nickel-base superalloys Part I: Ostwald ripening theories. J. Mater. Sci..

[31] Chen Y.H., Doherty R.D. (1977). On the growth kinetics of plate-shaped precipitates in aluminium-copper and aluminium-gold alloys. Scripta Metall..

[32] Liu G., Zhang G.J., Ding X.D., Sun J., Chen K.H. (2003). Modeling the strengthening response to aging process of heat-treatable aluminum alloys containing plate/disc- or rod/needle-shaped precipitates. Mater. Sci. Eng. A.

[33] Vaithyanathan V., Wolverton C., Chen L.Q. (2004). Multiscale modeling of θ′ precipitation in Al–Cu binary alloys. Acta Mater..

[34] Rakhmonov J., Timelli G., Bonollo F. (2016). The Effect of Transition Elements on High-Temperature Mechanical Properties of Al–Si Foundry Alloys–A Review. Adv. Eng. Mater..

[35] Medrano S. (2018). Study of Concurrent Recovery and Precipitation on the Mechanical Behaviour of Al-Mg alloys with Small Additions of Cu. Doctoral Dissertation.

[36] Jiang S., Wang R. (2019). Grain size-dependent Mg/Si ratio effect on the microstructure and mechanical/electrical properties of Al-Mg-Si-Sc alloys. J. Mater. Sci. Technol..

[37] Li Z., Zhang Z., Chen X.-G. (2018). The Influence of Cu Addition on Dispersoid Formation and Mechanical Properties of Al-Mn-Mg 3004 Alloy. Metals.

[38] Mei X.M., Mei Q.S., Peng Y.Q., Chen Z.H., Xu T., Wang Y.C. (2022). Achieving enhanced mechanical properties of SiC/Al–Cu nanocomposites via simultaneous solid-state alloying of Cu and dispersing of SiC nanoparticles. Mater. Sci. Eng. A.

